# Characterization of Oyster Voltage-Dependent Anion Channel 2 (VDAC2) Suggests Its Involvement in Apoptosis and Host Defense

**DOI:** 10.1371/journal.pone.0146049

**Published:** 2016-01-04

**Authors:** Yingxiang Li, Linlin Zhang, Tao Qu, Li Li, Guofan Zhang

**Affiliations:** 1 University of Chinese Academy of Sciences, Beijing, China; 2 Laboratory for Marine Biology and Biotechnology, Qingdao National Laboratory for Marine Science and Technology, Qingdao, China; 3 Key Laboratory of Experimental Marine Biology, Institute of Oceanology, Chinese Academy of Sciences, Qingdao, China; 4 National & Local Joint Engineering Laboratory of Ecological Mariculture, Institute of Oceanology, Chinese Academy of Sciences, Qingdao, China; Institute of Oceanology, Chinese Academy of Sciences, CHINA

## Abstract

Genomic and transcriptomic studies have revealed a sophisticated and powerful apoptosis regulation network in oyster, highlighting its adaptation to sessile life in a highly stressful intertidal environment. However, the functional molecular basis of apoptosis remains largely unexplored in oysters. In this study, we focused on a representative apoptotic gene encoding voltage-dependent anion channel 2 (VDAC2), a porin that abounds at the mitochondrial outer membrane. This is the first report on the identification and characterization of a VDAC gene in the Pacific oyster, *Crassostrea gigas* (*CgVDAC2*). The full length of *CgVDAC2* was 1,738 bp with an open reading frame of 843 bp that encoded a protein of 281 amino acids. A four-element eukaryotic porin signature motif, a conserved ATP binding motif, and a VKAKV-like sequence were identified in the predicted CgVDAC2. Expression pattern analysis in different tissues and developmental stages as well as upon infection by ostreid herpesvirus 1 revealed the energy supply-related and immunity-related expression of *CgVDAC2*. CgVDAC2 was co-localized with mitochondria when it was transiently transfected into HeLa cells. Overexpression of CgVDAC2 in HEK293T cells suppressed the UV irradiation-induced apoptosis by inhibiting the pro-apoptotic function of CgBak. RNA interference induced reduction in *CgVDAC2* expression showed a promoted apoptosis level upon UV light irradiation in hemocytes. The yeast two-hybrid system and co-immunoprecipitation assay indicated a direct interaction between CgVDAC2 and the pro-apoptotic protein CgBak. This study revealed the function of VDAC2 in oyster and provided new insights into its involvement in apoptosis modulation and host defense in mollusks.

## Introduction

As an estuarine and intertidal zone animal with sessile behavior, oyster is exposed to fluctuating temperature, variable salinity, toxic metals, and desiccation, which are highly stressful conditions for this sedentary organism [1]. Microbial pathogens are also serious challenges for the filter-feeding oysters living in pathogen-rich seawaters. The successful adaptation to the environment makes oyster an attractive model for studying the relationship of immune and stress adaptation [2].

Apoptosis plays a key role in immune and stress defense in mollusks, mainly by limiting the spread of parasites and pathogens and preventing the inflammatory damage of surrounding tissues [1,3]. Mitochondrial apoptosis is a vital type of apoptosis that functions in host defense process. *CgBcl-2*, *CgIAP*, *CgBax* and *CgBak* are all known as the kernel elements of this pathway in mollusks. Previous reports indicated that these genes are relevant to host protection against parasites and pathogens such as ostreid herpesvirus 1 (OsHV-1), *Vibrio alginolyticus*, *Staphylococcus haemolyticus*, *Saccharomyces cerevisiae*, *Vibrio anguillarum* and *Roseovarius crassostreae* [4–9]. In mollusks, interactions between immune cells and parasites or pathogens usually trigger apoptosis, nevertheless, some pathogens are capable to suppress this host response after infection [10]. However, the underlying mechanisms of the pathogen-induced regulation of apoptosis in mollusks remain unclear [11].

The voltage-dependent anion channel (VDAC), first identified in *Paramecium aurelia*, is a porin, mainly located at the mitochondrial outer membrane (MOM), which offers a predominant pathway for the diffusion of ions and metabolites across MOM [12–14]. VDAC is proposed as part of the mitochondrial permeability transition pores composed of adenine nucleotide translocase and cyclophilin D [13,15,16]. The pore or channel enables the exchange of metabolites between the cytosol and mitochondria. Therefore, VDAC plays a pivotal role in the modulation of metabolic and energy functions in mitochondria [17–20]. It has been reported that VDAC is involved in numerous pathological conditions, which may result from the disturbance of VDAC function in energy production, metabolite crosstalk between the cytosol and mitochondria, or apoptosis regulation [15]. Although it is not clear whether the regulation of VDAC in the host cell is the key for these pathological conditions, previous studies have revealed the involvement of VDAC in the mitochondria-mediated apoptosis pathway [21–23] via its interaction with various apoptosis-related proteins and the regulation of mitochondrial proteins such as cytochrome c [13,24–27,16].

Various pathogenic organisms, including viruses, bacteria, and protists, are constant threats to the health of aquatic animals globally. Previous studies that focused on host defense genes have provided basic knowledge on the mechanism against pathogens, however, it remains unknown whether and how VDAC is involved in the response immune system of aquatic animals. In fish and shrimp, up-regulation of VDAC under viral infection has been reported [28,29], but the underlying molecular mechanism remains unclear. Additionally, the function of VDAC in bivalve mollusks remains unexplored.

In the present study, we identified a VDAC2 homologous gene (*CgVDAC2*) in *C*. *gigas*, a commercially important aquatic bivalve mollusk, and characterized its protein structure elements. We subsequently investigated the expression profiles of *CgVDAC2* in different tissues, developmental stages and upon infection by OsHV-1. Then, we studied the subcellular localization of CgVDAC2 in HeLa cells and its anti-apoptotic function in HEK293T cells and oyster hemocytes. We also examined the signal transduction between CgVDAC2 and the pro-apoptotic protein CgBak. This study aimed to provide insight into various biological functions of CgVDAC2, mainly focusing on the immune and apoptotic functions and the defense system of *C*. *gigas*.

## Materials and Methods

### Ethics Statement

The Pacific oyster, *C*. *gigas*, used in this study is a marine-cultured animal and cultured in the aquarium at Institute of Oceanology, Chinese Academy of Sciences (IOCAS). All of the experiments were conducted according to local and national regulations. No specific permissions were required for the oyster sample collection and described experiments. All of the field studies were carried out at IOCAS, and did not involve any endangered or protected species.

### Animal materials, tissue and virus collection, and treatments

Pacific oysters used in this study were obtained from Qingdao, Shangdong Province, China, and maintained at 22 ± 1°C in tanks with circulating seawater for 7 d prior to the treatment.

Equivalent amounts of tissue from mantles, gills, female gonads, male gonads, adductor muscles, digestive glands, labial palps, and the hemolymph were sampled from six healthy oysters and pooled for RNA extraction and tissue-specific expression analysis. Typical larval samples from 11 different developmental stages (fertilized-egg, two-cell, four-cell, morula, blastula, gastrula, trochophore, early D-shape larval, late D-shape larval, early umbo larval and late umbo larval stages) were collected [1].

OsHV-1 was isolated from naturally infected oysters that were ground, filtered, and centrifuged. OsHV-1 was detected and quantified in the supernatant using quantitative polymerase chain reaction (qPCR) with the primers C9/C10 (Table 1). Then, phosphate-buffered saline (PBS) was added to the supernatant to adjust the concentration of OsHV-1 to approximately 10^6^ ml^-1^. A 100-μL sample was injected to the adductor muscle of each oyster using a 1-ml syringe. An equal volume of PBS was used as a negative control. After the injection, oysters were placed back into the seawater tanks and the hemolymphs of five random individuals were collected at 0, 3, 6, 12, 24, 48, and 72 h post injection (hpi). Hemolymph samples were centrifuged and stored in TRIzol (Invitrogen, Carlsbad, CA, USA), according to the manufacturer’s instructions.

**Table 1:** The primer and siRNA sequences used in the study.

Sequence ID	Sequences (5'–3')	Application
CgVDAC2F1	CCCTTGTGGACGGCAAGAACT	3' RACE^a^
CgVDAC2F2	GGGTCTCGGTCTGGACTTCG	
CgVDAC2R1	TGTCACTGTGACTGCCTGTTGT	5' RACE
CgVDAC2R2	GAACACACGCTGAAGGTCACAATCT	
Oligo(dT)-adaptor	GGCCACGCGTCGACTAGTACT_16_	RACE
Oligo(dG)-adaptor	GGCCACGCGTCGACTAGTACG_10_	
adaptor	GGCCACGCGTCGACTAGTAC	
CgVDAC2qF	TATTCCGACTTAGGTCTTACAT	qPCR^b^
CgVDAC2qR	CAGTTTCAATCCTTTCACG	
CgRS18F	GCCATCAAGGGTATCGGTAGAC	
CgRS18R	CTGCCTGTTAAGGAACCAGTCAG	
CgEFF	TTGTTGTTGACTGCGTATCTGGTGT	
CgEFR	GGGTTGTCTTCGATTCCATAGGTAG	
CgGAPDHF	TTCTCTTGCCCCTCTTGC	
CgGAPDHR	CGCCCAATCCTTGTTGCTT	
CgVDAC2-MycF	CATGGAGGCCCGAATTATGGCTCCCCCAACATATGGTGA	co-IP assay^c^
CgVDAC2-MycR	CTCGGTCGACCGAATTTCAGGCCTCGAAGTCCAGACCG	
CgBak-FlagF	CTCAAGCTTCGAATTCTGATGGCTCCCCCAACATATGGTGA	
CgBak-FlagR	GTCGACTGCAGAATTCGTCAGGCCTCGAAGTCCAGACCG	
CgVDAC2-EGFPF	CTCAAGCTTCGAATTCTGATGGCTCCCCCAACATATGGTGA	Subcellular localization and caspase3 activity assay
CgVDAC2-EGFPR	GTCGACTGCAGAATTCGTCAGGCCTCGAAGTCCAGACCG	
CgVDAC2-pGADF	GGAGGCCAGTGAATTCATGGCTCCCCCAACATATGGTGATCT	Yeast two-hybrid system
CgVDAC2-pGADR	CGAGCTCGATGGATCCCGAAGTCCAGACCGAGA	
CgBak-pGBKF	CATGGAGGCCGAATTCATGGCTTACTGGGACGGTGGT	
CgBak-pGBKR	GCAGGTCGACGGATCCACTCGCTGGACTTCAACTCTTTT	
CgBak-EGFPF	CTCAAGCTTCGAATTCTGATGGCTTACTGGGACGG	Caspase3 activity assay
CgBak-EGFPR	GTCGACTGCAGAATTCGCGACCCCACAACAATGGA	
C9	GAGGGAAATTTGCGAGAGAA	Quantity of OsHV-1
C10	ATCACCGGCAGACGTAGG	
CgVDAC2-siRNA-188 sense	CCGACUUAGGUCUUACAUUTT	RNAi assay^d^
CgVDAC2-siRNA-188 anti-sense	AAUGUAAGACCUAAGUCGGTT	
CgVDAC2-siRNA-264 sense	GCUCGUGAAAGGAUUGAAATT	
CgVDAC2-siRNA-264 anti-sense	UUUCAAUCCUUUCACGAGCTT	
CgVDAC2-siRNA-530 sense	CCAUUCACACCAAUGUCAATT	
CgVDAC2-siRNA-530 anti-sense	UUGACAUUGGUGUGAAUGGTT	
Negative Control siRNA sense	UUCUCCGAACGUGUCACGUdTdT	
Negative Control siRNA anti-sense	ACGUGACACGUUCGGAGAAdTdT	

^a^ RACE, rapid amplification of cDNA ends

^b^ qPCR, quantitative polymerase chain reaction

^c^ Co-IP, co-immunoprecipitation

^d^ RNAi, RNA interference

### Characterization of the full-length cDNA sequence of *CgVDAC2*

The coding sequence (CDS) of *CgVDAC2* was downloaded from the OysterBase (http://www.oysterdb.com) and used to design and synthesize primers for CDS amplification. After the validation of CDS, primers for the rapid amplification of cDNA ends (RACE) were designed and synthesized. The 3' end of *CgVDAC2* was cloned using the 3' RACE system (Invitrogen, Carlsbad, CA, USA), according to the manufacturer’s instructions, with gene specific primers (CgVDAC2F1 and CgVDAC2F2) and an Oligo(dT)-adaptor (Table 1). After the addition of a dCTP tail to cDNA using the terminal transferase TdT (Invitrogen, Carlsbad, CA, USA), according to the manufacturer’s instructions, the 5' end of *CgVDAC2* was cloned with gene specific primers (CgVDAC2R1 and CgVDAC2R2) and an Oligo(dG)-adaptor (Table 1). The open reading frame (ORF) was predicted by the ORF Finder in the National Center for Biotechnology Information (NCBI) database (http://www.ncbi.nlm.nih.gov/projects/gorf/) using the full-length cDNA sequence acquired by the combination of 3'-end sequence, 5'-end sequence, and validated CDS.

The deduced amino acid sequence was obtained by Primer Premier 5 (Premier Biosoft, Palo Alto, CA, USA) and analyzed using BLAST in NCBI (http://blast.ncbi.nlm.nih.gov/Blast.cgi). The amino acid sequence of CgVDAC2 was aligned with representative invertebrate and vertebrate VDAC proteins using Mega5 (http://www.megasoftware.net). The molecular weight and theoretical isoelectric point of the predicted protein was calculated using ProtParam tool (http://web.expasy.org/protparam/).

### Total RNA isolation and transcriptional analysis of *CgVDAC2*

Total RNA was extracted from each sample using 1 ml TRIzol (Invitrogen, Carlsbad, CA, USA), and its integrity was assessed using agarose gel electrophoresis. To construct the cDNA, 1 mg of total RNA was reverse-transcribed using PrimeScript RT reagent kit with gDNA Eraser (TaKaRa, Shiga, Japan), according to the manufacturer’s instructions. Then, qPCR was performed using ABI 7500 Fast Real-Time PCR System (Applied Biosystems, Foster City, CA, USA), in a 20 μL volume, consisting of 10 μL of 2X SYBR premix Ex Taq (TaKaRa, Shiga, Japan), 0.4 μL of each primer (10mM), 0.4 μL 50X Rox reference dye, 3 μL oyster cDNA template, and 5.8 μL RNase-free water. Gene-specific primers for *CgVDAC2* amplification are listed in Table 1. PCR conditions were as follows: 95°C for 30 s, followed by 40 cycles of 95°C for 5 s and 60°C for 30 s. A melting curve analysis was run to confirm the specificity of the amplicons. Each sample was analyzed in triplicate. Data were analyzed using 7500 software v2.0.1 (Applied Biosystems, Foster City, CA, USA).

CgRS18 primers, Cg Elongation factor (EF) primers, and CgGAPDH primers were used as internal controls in the expression pattern analysis of different developmental stages, different tissues, and upon infection by OsHV-1, respectively (Table 1), as described by Du et al. [30] and Zhang et al. [7]. In RNA interference (RNAi) assays, CgGAPDH primers were selected as internal controls. The transcript level of each gene was normalized to the expression of their respective internal controls and the comparative 2^-ΔΔCq^ method was used to calculate the gene expression of the samples [31].

### Plasmid construction

The full-length cDNAs of *CgVDAC2* and *CgBak* were sub-cloned using In-Fusion HD Cloning Kit (TaKaRa, Shiga, Japan) into the mammalian expression vectors pCMV-N-Myc and pCMV-N-Flag (Beyotime, Jiangsu, China), respectively, according to the manufacturer’s instructions. To investigate the subcellular localization of CgVDAC2, the pEGFP-N1-CgVDAC2 plasmid was constructed using In-Fusion HD Cloning Kit (TaKaRa, Shiga, Japan). The pEGFP-N1-CgBak plasmid was also constructed using the same kit for the overexpression experiments in HEK293T cells. In addition, *CgVDAC2* and *CgBak* were sub-cloned into the two-hybrid plasmids pGADT7 and pGBKT7 (TaKaRa, Shiga, Japan), respectively, and used in the yeast two-hybrid system.

### Cell culture and plasmid transfection

The primary Pacific oyster hemocyte culture was carried out as described by Yu [32] with some modifications. Briefly, the hemocytes were withdrawn from the pericardial cavity using a sterile 21-gauge needle that was attached to a 1-ml syringe containing 0.1–0.2 ml chilled filtered sterile seawater (FSW) supplemented with 2 g L^-1^ glucose. The cells were then plated into 6-well plates, their concentration was adjusted to approximately 2 × 10^6^ per well, and allowed to attach for 30 min at room temperature. Thereafter, 2 ml Leibovitz L-15 medium (Sigma Aldrich, St. Louis, MO, USA) supplemented with 0.54 g L^-1^ KCl, 20.2 g L^-1^ NaCl, 0.6 g L^-1^ CaCl_2_, 3.9 g L^-1^ MgCl_2_, 1 g L^-1^ MgSO_4_, 20.8 g L^-1^ glucose, 200 μg ml^-1^ streptomycin, 100 μg ml^-1^ gentamycin, and 10% fetal bovine serum (Hyclone, Logan, UT, USA) was added to suspend the cells immediately after the hemolymph was removed. The plates were incubated at 18°C.

For the culture of mammal cells, HEK293T cells (ATCC, Manassas, VA, USA) were cultured in Dulbecco’s Modified Eagle’s Medium (DMEM)/High Glucose (HyClone, Logan, UT, USA), while HeLa cells were cultured in modified Roswell Park Memorial Institute (RPMI)-1640 medium (HyClone, Logan, UT, USA). Both media were supplemented with 10% fetal bovine serum (HyClone, Logan, UT, USA), penicillin (100 U ml^-1^), and streptomycin (100 U ml^-1^). Cells were maintained at 37°C in 5% CO_2_. The plasmids were transiently transfected into cells using Lipofectamine 3000 (Invitrogen, Carlsbad, CA, USA) according to the manufacturer’s instructions. Then, pEGFP-N1-CgVDAC2 was transfected into HeLa cells, while plasmids for the co-immunoprecipitation (co-IP) assay and overexpression assay were transfected into HEK293T cells.

### Subcellular localization and co-IP assay

For subcellular localization analysis, HeLa cells transfected with pEGFP-N1-CgVDAC2 or pEGFP-N1 were rinsed once with PBS at 24 h post transfection; stained with 2 mg ml^-1^ Hoechst33342 (Invitrogen, Carlsbad, CA, USA) that dissolved in PBS for 10 min at 37°C; rinsed twice with PBS; stained with Alexa Fluor 594 (Life Technologies, Carlsbad, CA, USA) for 15 min at 37°C; rinsed three times with PBS; maintained in modified RPMI-1640 medium without fetal bovine serum; and visualized by confocal microscopy (Carl Zeiss, Oberkochen, Germany).

HEK293T cells were divided into two groups, a group that co-transfected by pCMV-N-Myc-CgVDAC2 and pCMV-N-Flag-CgBak and the other group that co-transfected by pCMV-N-Myc and pCMV-N-Flag-CgBak. Cells were lysed in RIPA Lysis Buffer (Beyotime, Jiangsu, China) at 4°C for 30 min in the presence of 1 mM phenylmethylsulfonyl fluoride (Beyotime, Jiangsu, China) and Protease Inhibitor Cocktail (ComWin Biotech, Beijing, China). Lysates were then centrifuged at 12,000 rpm for 5 min at 4°C. The supernatant was collected, and separated into two parts: one was stored as the input sample and the remaining was mixed with ANTI-FLAG M2 Magnetic Beads (Sigma-Aldrich, St. Louis, MO, USA) and shaken gently on a roller shaker for 2 h. Then, the beads were washed three times with cell lysis buffer and incubated with 2X Protein sodium dodecyl sulfate polyacrylamide gel electrophoresis (SDS PAGE) loading buffer (TaKaRa, Shiga, Japan) at 100°C for 5 min. Proteins in the loading buffer (IP samples) were analyzed using western blotting.

### RNAi and UV light irradiation of hemocytes and determination of hemocyte apoptosis levels

Small interfering RNAs (siRNAs) targeting *CgVDAC2* were synthesized by GenePharma (Shanghai, China) based on the sequence of the gene. A pool of three distinct siRNAs (Table 1), all targeting Cg*VDAC2*, was used in this RNAi assay. A total of 50 μg siRNA of *CgVDAC2* or solely siRNA that was used as a negative control (Table 1) or no siRNA was dissolved in 50 μl PBS and gently added to each well. A part of the hemocytes was collected after 24 h to detect the expression level of *CgVDAC2* in each group using qPCR. The rest were irradiated by UV light for 20 min. The apoptosis level of the hemocytes was measured 20 h after UV light irradiation using FITC Annexin V Apoptosis Detection Kit (BD Biosciences, San Jose, CA, USA) following the manufacturer’s instructions. The samples were analyzed using a FACS Calibur flow cytometer (BD, San Jose, CA, USA), and 10,000 events were counted for each hemocyte sample.

### Activity assays of caspase3 in CgBak and CgVDAC2 overexpressed HEK293T cells

Recombinant CgBak and CgVDAC2 with EGFP-tag were expressed in HEK293T cells, respectively. The expressions of both proteins were detectable by Western Blot with anti-GFP monoclonal antibody. Then, HEK293T cells were transfected with distinct plasmids. 16 h later, the cells were irradiated by UV-light for 15 min (while the control cells not) and harvested 24 h after the UV-irradiation. The total protein was extracted and concentration of it was measured using the Bradford method for activity assay of caspase3. Caspase 3 Activity Assay Kit (Beyotime, Jiangsu, China) was used for the assay, and all the manipulation followed the protocol provided by the company.

### Yeast two-hybrid system

The yeast two-hybrid system was carried out to study the interaction between CgVDAC2 and CgBak using Clontech Matchmaker Gold Yeast Two-Hybrid System (TaKaRa, Shiga, Japan). pGADT7-CgVDAC2 and pGBKT7-CgBak were transformed into the Y187 and Gold yeast strains, respectively. Y187 cells were cultured onto selective plates with synthetically defined medium (SD) lacking leucine (SD/-Leu), while Gold cells were cultured on SD plates lacking tryptophan (SD/-Trp). After 3 d, positive yeast strains on SD/-Leu and SD/-Trp were hybridized in 2X yeast extract peptone dextrose (YPDA) medium and selected on double drop-out SD/-Leu/-Trp medium. The interaction between CgVDAC2 and CgBak was detected by the hybridized clones growing on quadruple drop-out SD/-Ade/-His/-Leu/-Trp medium supplemented with X-α-Gal and Aureobasidin A (TaKaRa, Shiga, Japan).

### Statistical analysis

All experiments were performed in triplicate, and data were analyzed by one-way analysis of variance (ANOVA) followed by a multiple comparison using the SPSS software package. The differences were considered statistically significant at *P* < 0.05.

## Results

### Sequence analyses of *CgVDAC2*

**Fig 1:**
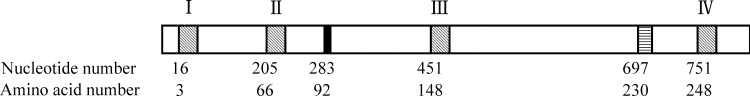
Small motif architecture of *VDAC2* in *Crassostrea gigas*. Motif analysis of *CgVDAC2*. *CgVDAC2* contains a 4-element eukaryotic porin signature motif (box with diagonal lines), a glycine-leucine-lysine (GLK) motif (black box), and a VKAKV-like sequence (box with horizontal lines). The four-element eukaryotic porin signature motif is indicated above. The nucleotide and amino acid number of the start site of each motif is labeled below.

The full-length cDNA sequence of *CgVDAC2* consists of an ORF of 843 bp, a 5' untranslated region (UTR) of 39 bp, and a 3' UTR of 856 bp with a poly (A) tail (S1 Fig). The predicted protein consisted of 280 amino acids (S1 Fig), with a deduced molecular weight (Mw) of 30.33 kDa and a theoretical isoelectric point of 8.25. Functional motif architecture analysis showed the existence of a porin3 domain, which is a distinctive characteristic of pore-forming proteins. In addition, the *CgVDAC2* coding region contained a four-element eukaryotic porin signature motif, a glycine-leucine-lysine (GLK) motif that is a presumed ATP binding site [33–35], and a VKAKV-like sequence (Fig 1), which is probably involved in the protein incorporation into MOM.

### Comparative analysis of *CgVDAC2*

A multiple sequence alignment of *VDAC2* from five vertebrate and five invertebrate species was conducted based on the deduced amino acid sequences and showed that *VDAC2* sequences were strongly homologous and conserved. The peptide sequence of CgVDAC2 displayed a strong identity with VDAC2 from *Homo sapiens* (61.79%), *Mus musculus* (61.43%), *Danio rerio* (63.93%), *Xenopus laevis* (63.93%), *Drosophila melanogaster* (60.36%), *Lepeophtheirus salmonis* (49.29%), *Aplysia californica* (63.93%), *Haliotis diversicolor* (60.36%), *Hydra vulgaris* (48.21%), and *Nematostella vectensis* (56.43%) (S2 Fig), showing that CgVDAC2 might have similar function as that in other model organisms.

**Fig 2:**
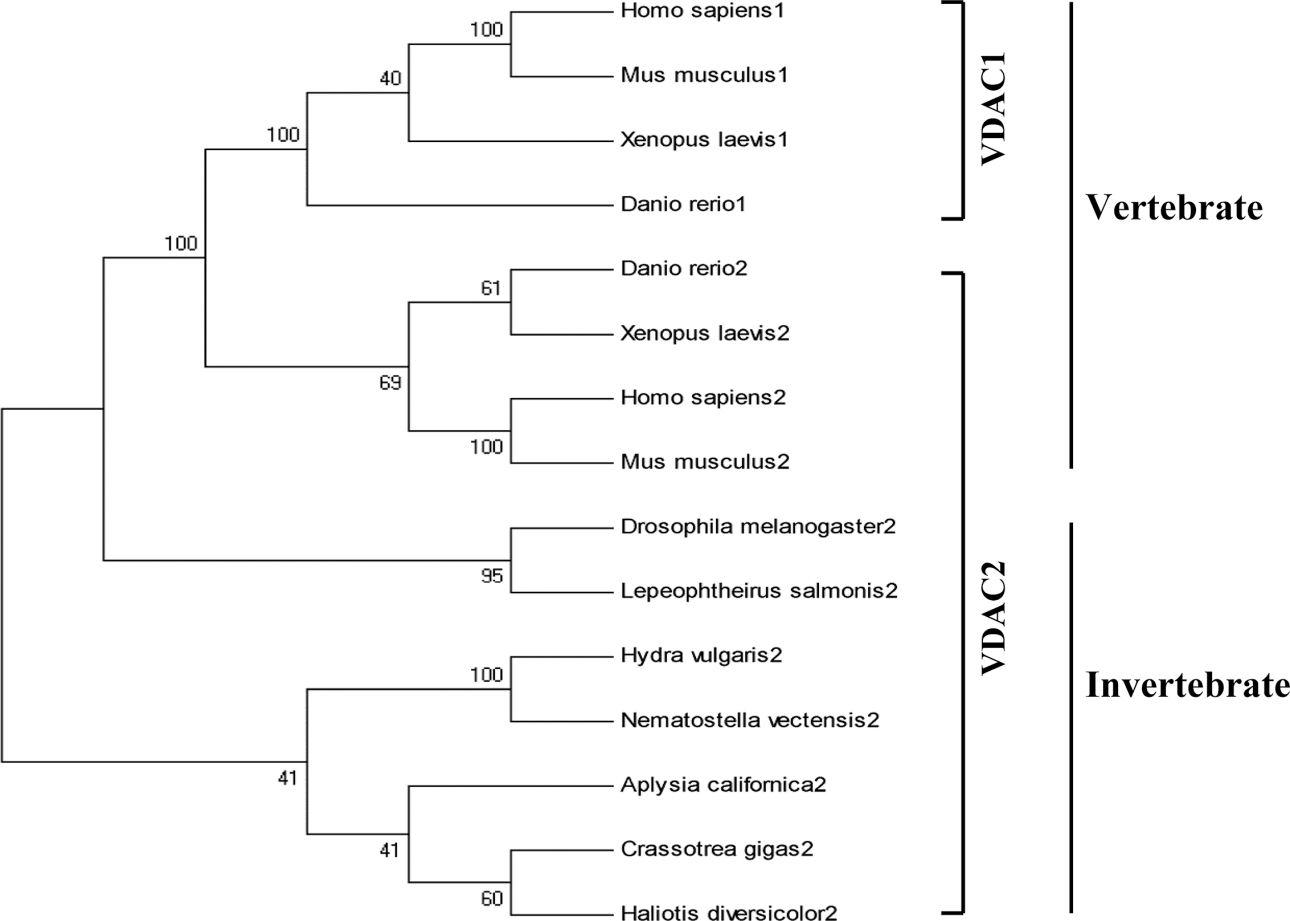
Neighbor-joining phylogenetic tree of *VDAC1*s and *VDAC2*s from different vertebrate and invertebrate species. The neighbor-joining tree which was built by MEGA program was based on the sequences of *CgVDAC2* from *Crassostrea gigas*, and *VDAC2*s from other species, including *Homo sapiens* (NP_003366), *Mus musculus* (NP_035825), *Xenopus laevis* (NP_001089399), *Danio rerio* (NP_955879), *Aplysia californica* (XP_005113380), *Haliotis diversicolor* (ADI56517), *Drosophila melanogaster* (NP_609462), *Lepeophtheirus salmonis* (ADD24283), *Nematostella vectensis* (XP_001623935), *Hydra vulgaris* (XP_002167561). Besides, four VDAC1 sequences from *Homo sapiens* (NP_003365), *Mus musculus* (NP_035824), *Xenopus laevis* (NP_001080684), and *Danio rerio* (NP_001001404) were also used in the phylogenetic analysis.

Phylogenetic analysis revealed that *CgVDAC2* was clustered into the mollusk branch, close to the sequences from cnidarians. The sequences from mollusks were more homologous with those from arthropods, instead of vertebrates (Fig 2). In addition, *CgVDAC2* was closer to the sequences of *VDAC2* from vertebrates, than the sequences of *VDAC1* from invertebrates, suggesting its ancient origin.

### Expression pattern analysis of *CgVDAC2*

**Fig 3:**
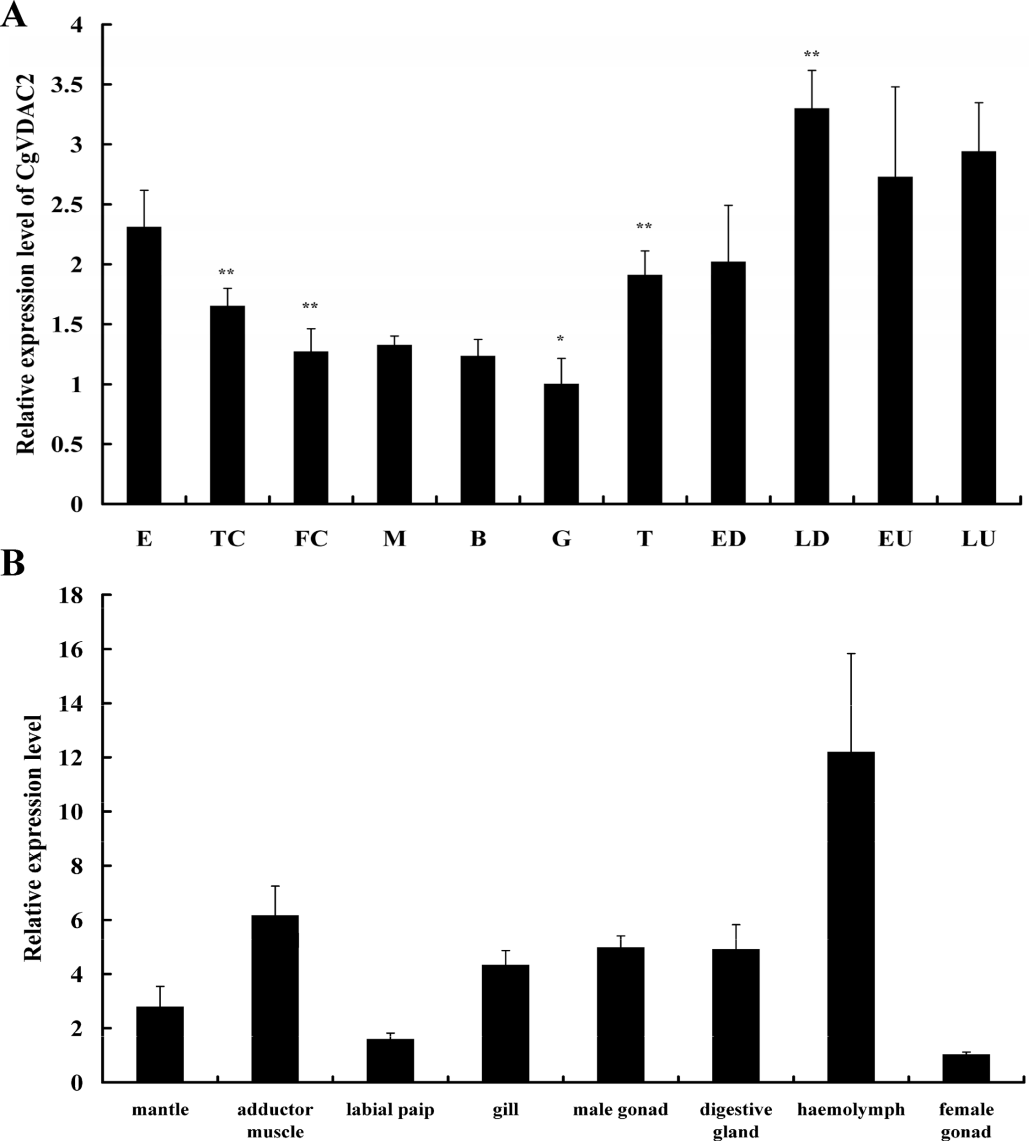
Developmental stage distributions and tissue distributions of *CgVDAC2* transcripts. (A) *CgVDAC2* mRNA expression pattern at 11 different developmental stages of *Crassostrea gigas*. Data are displayed as the mean ± standard error of triplicate independent experiments. E, egg stage; TC, two-cell stage; FC, four-cell stage; M, morula stage; B, blastula stage; G, gastrula stage; T, trochophore stage; ED, early D-shape larval stage; LD, late D-shape larval stage; EU, early umbo larval stage; LU, late umbo larval stage. Significant differences between the expression levels in each developmental stage and those in the former stage were identified using *t*-test. Asterisks indicate significant differences at *P* < 0.05 * and *P* < 0.01**. **(**B) *CgVDAC2* mRNA expression pattern in eight different tissues of *Crassostrea gigas*. *CgVDAC2* expression in mantle, adductor muscle, labial palp, gill, male gonad, digestive gland, hemolymph and female gonad are shown. Data are displayed as the mean ± standard error of triplicate independent experiments. Asterisks indicate significant differences at *P* < 0.05 * and *P* < 0.01**.

The transcription level of *CgVDAC2* was relatively high at the egg stage, slightly decreased from the two-cell to the trochophore stage, and then increased and reached a peak at the late D-shape larval stage (Fig 3A). *CgVDAC2* mRNA expression was detectable in all the eight sampled tissues. Relatively high transcription levels were detected in the hemolymph, adductor muscle, male gonad, digestive gland, and gill (Fig 3B). Relatively low transcription levels were detected in the female gonad, labial palp, and mantle (Fig 3B).

**Fig 4:**
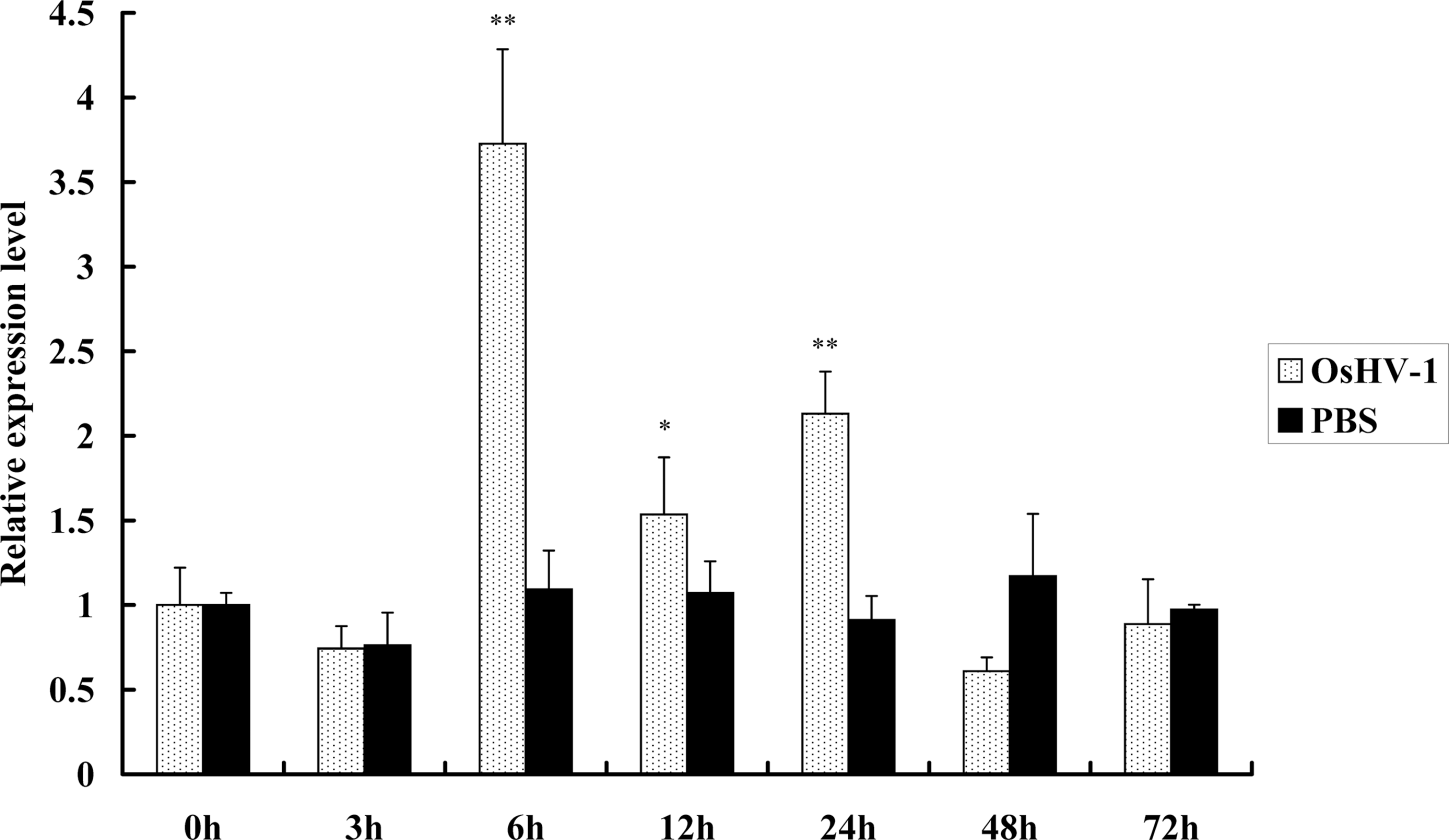
Expression pattern of *CgVDAC2* transcript in ostreid herpesvirus 1 (OsHV-1) infected hemolymph. Quantitative PCR analysis of *CgVDAC2* expression in *C*. *gigas* at 0, 3, 6, 12, 24, 48 and 72 h after treatment with OsHV-1 (infected samples) or phosphate-buffered saline (PBS, control). Samples at 0 h after both treatments were used as the reference samples. The experiments were repeated three times. The values are shown as the mean ± S.D (N = 3). Asterisks indicate significant differences at *P* < 0.05 * and *P* < 0.01**.

*CgVDAC2* was significantly up-regulated in the hemolymph upon infection by OsHV-1. The transcription level of *CgVDAC2* was nearly 4-fold higher compared with the control at 6 hpi; then, it decreased and became approximately 1.5-fold higher compared with the control; it increased again at 24 hpi; and finally it decreased at a level similar to that of the control (Fig 4).

### CgVDAC2 is located on Mitochondrial Outer Membrane

**Fig 5:**
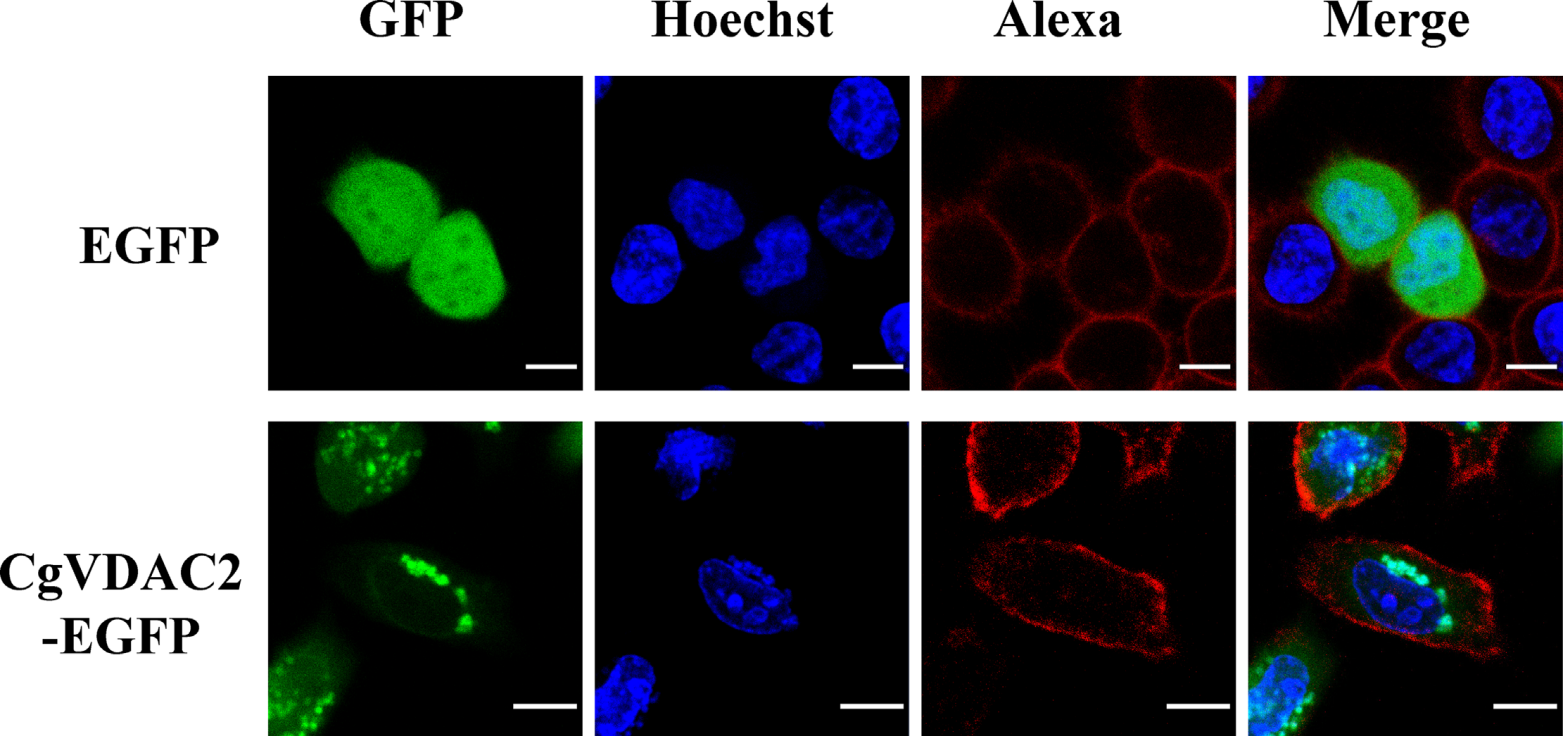
Subcellular localization of CgVDAC2-EGFP in HeLa cells. The plasmids of *CgVDAC2* and the enhanced green fluorescent protein (EGFP) negative control were transfected into HeLa cells (green). Cell nuclei are stained with Hoechst 33342 (blue) and cell membranes with Alexa Fluor 594 (red). The green fluorescent signal of CgVDAC2-EGFP is mainly localized to cytoplasmic puncta and indicates the co-localization of CgVDAC2 with mitochondria in HeLa cells. Scale bars = 5 μm.

In order to test whether *CgVDAC2* is translated on the mitochondrial outer membrane, pEGFP-N1-CgVDAC2 expression vector was constructed and transfected into HeLa cells, confocal laser scanning revealed that *CgVDAC2* co-localized with mitochondria in HeLa cells (Fig 5).

### CgVDAC2 inhibits UV irradiation-induced apoptosis

**Fig 6:**
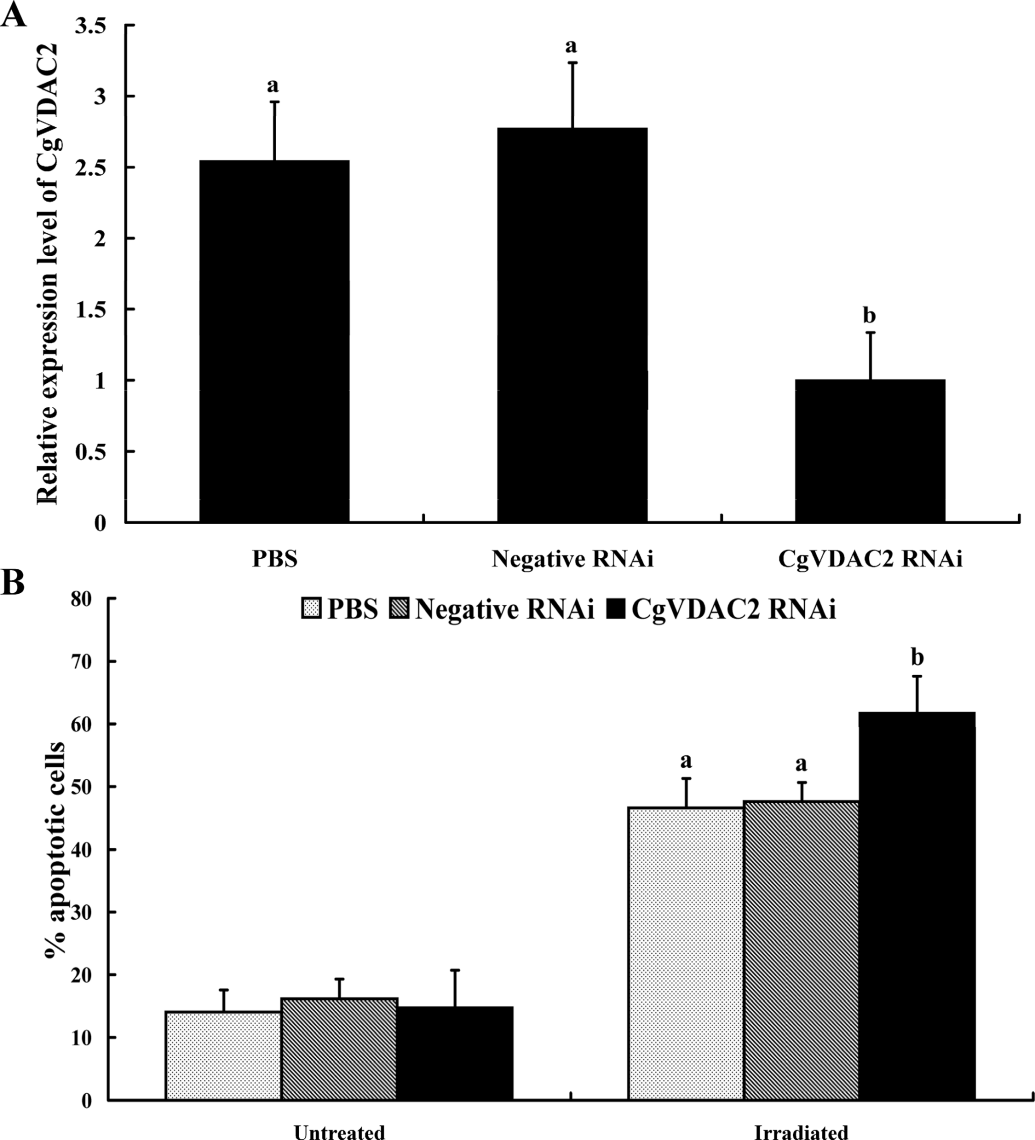
Effects of RNAi of *CgVDAC2* on UV irradiation-induced apoptosis in hemocytes of *C*.*gigas*. (A) Expression level of *CgVDAC2* in hemocytes of *C*.*gigas* after RNAi. Error bars represent standard error of three parallels. Different small letters indicate differences at *P* < 0.05. (B) Apoptosis level of hemocytes with RNAi-silenced *CgVDAC2* upon UV light irradiation. Values represent the mean ± SD of six samples. Different small letters indicate differences at *P* < 0.05.

CgVDAC2 RNAi was conducted to investigate its function in apoptosis. qPCR analysis showed that RNAi significantly suppressed the expression level of *CgVDAC2*. The mRNA expression level of *CgVDAC2* was down-regulated by 60.71% and 63.94% compared to that in the PBS treatment and the negative control, respectively (Fig 6A). Additionally, no statistical difference was identified between the transcription level of *CgVDAC2* in the PBS treatment and the negative control (Fig 6A). Upon UV irradiation, the hemocytes of *C*. *gigas*, with RNAi-silenced *CgVDAC2* showed a significantly higher apoptosis level (61.81%) compared with that in the PBS treatment (46.59%) and the negative control (47.59%) that had normal *CgVDAC2* expression levels (Fig 6B).

### CgVDAC2 interacts with the apoptosis-related protein CgBak

**Fig 7:**
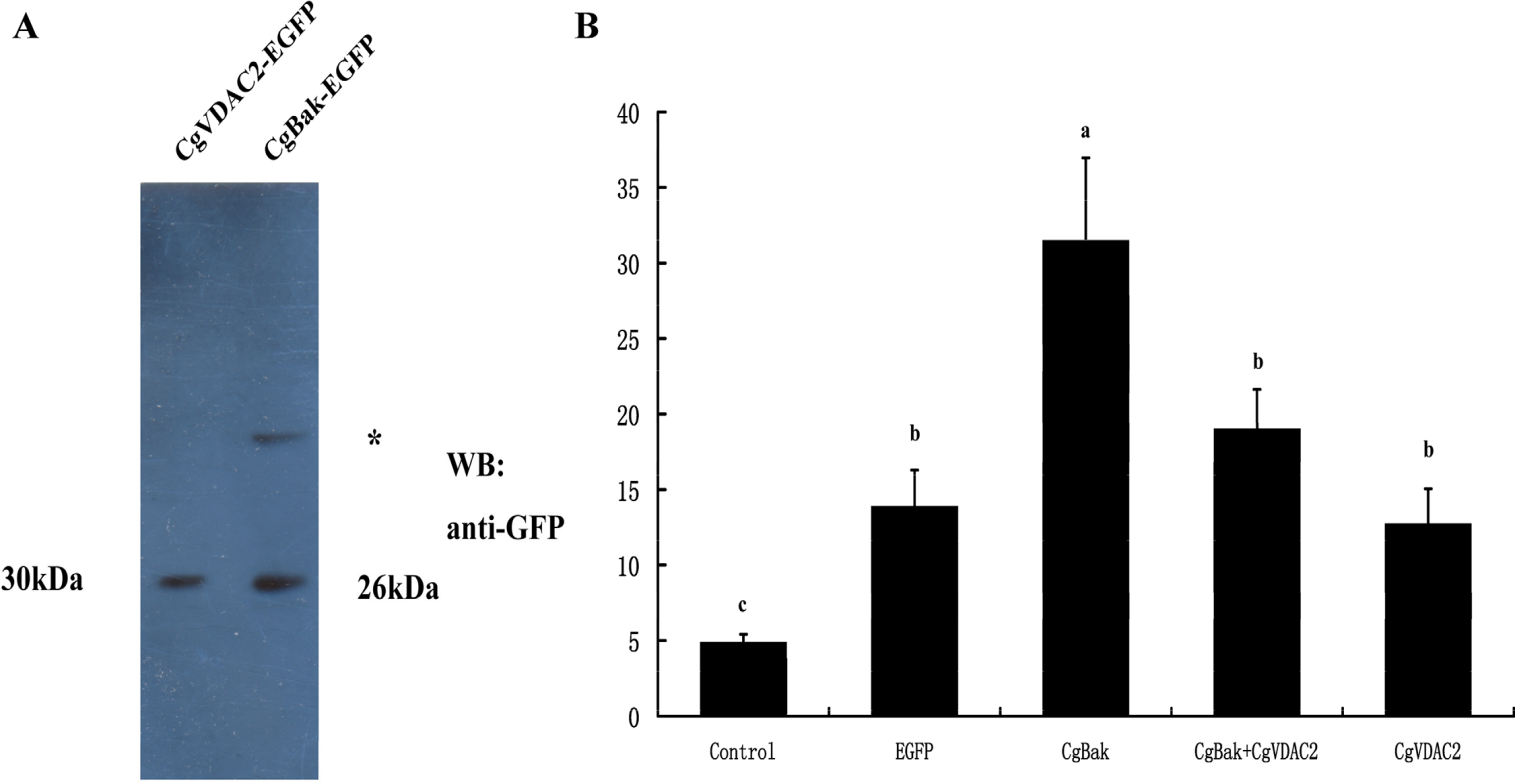
Effects of overexpression of CgVDAC2 on UV irradiation-induced apoptosis in HEK293T cells. (A) Recombinant expression of CgVDAC2 and CgBak in HEK293T cells. The deduced molecular weights of these two proteins are approximate 30 kDa and 26 kDa, respectively. The asterisk indicated a non-specific band. (B) Caspase3 activities of HEK293T cells that expressed distinct recombinant proteins. The Caspase3 activities were determined 24 h after UV irradiation and based on spectrophotometric detection of the chromophore *p*-nitroaniline (*p*NA) after cleavage from the labeled substrate DEVD-*p*NA. The values are shown as the mean ± S.D (N = 3). Different small letters indicate differences (*P* < 0.05) and the same letter indicated not.

In human cells, VDAC2 could regulate apoptosis by interacting with apoptosis-related protein Bak. We next aim to investigate whether similar mechanism exists in oyster. The increased Caspase 3 activities in CgBak overexpressed HEK293T cells were found to be inhibited by co-overexpression of CgVDAC2. After being irradiated by UV light, caspase3 activities in all the HEK293T cells increased compared with the activity in the control without treatment. Caspase3 activity in the CgBak-overexpression cells was the highest among the five groups while the activity in cells where CgBak and CgVDAC2 were co-overexpressed was remarkably lower than that, but appreciably higher than the activities in the other two UV irradiated groups (Fig 7).

**Fig 8:**
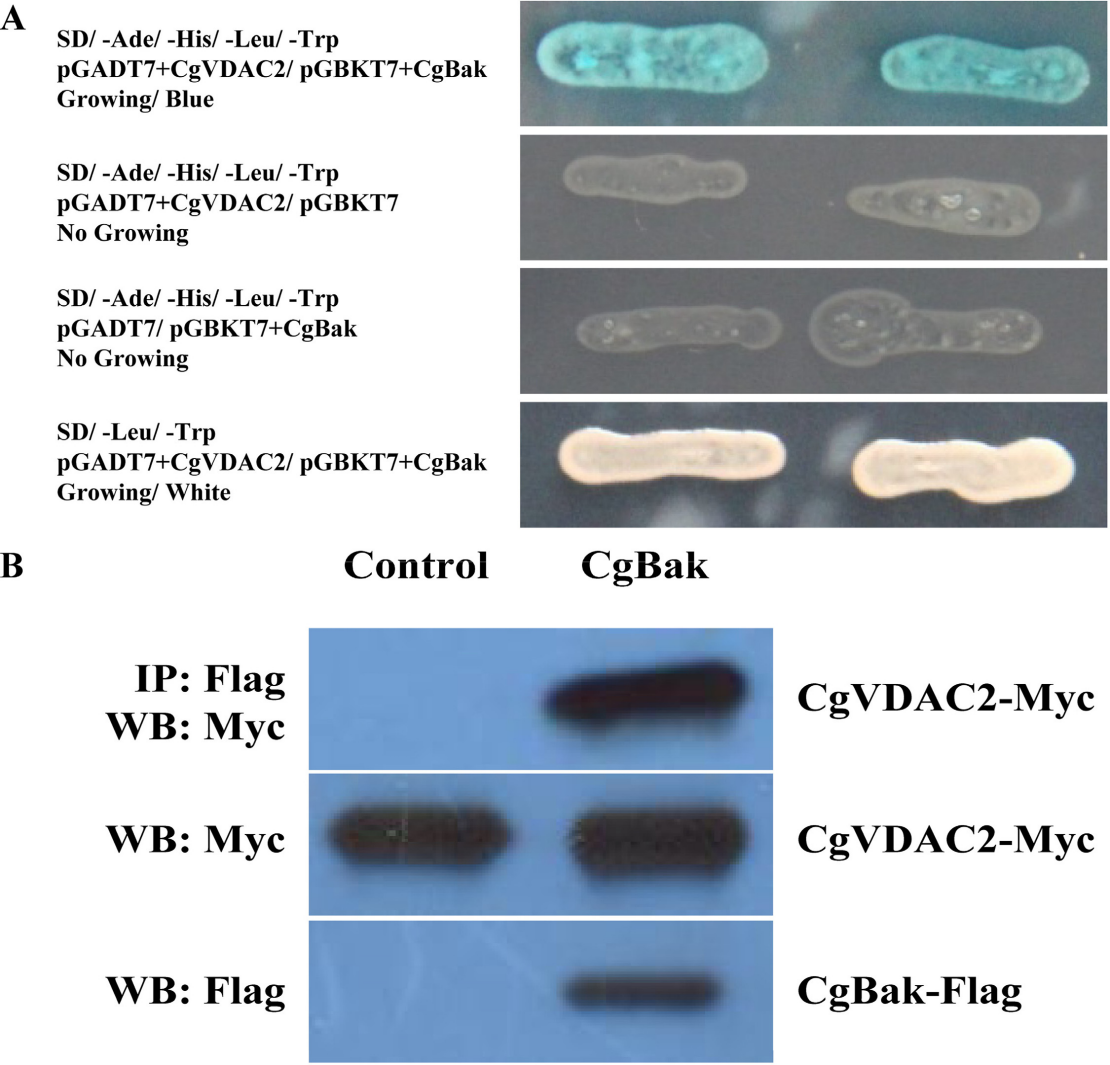
Interaction between CgVDAC2 and CgBak. (A) Interaction between CgVDAC2 and CgBak detected by the yeast two-hybrid system. The yeast was cultured on respective plates 3–5 days before observed and taken photos. The blue yeast colonies growing on quadruple drop-out SD/-Ade/-His/-Leu/-Trp medium indicated the direct interaction relation between the two proteins. (B) Interaction between CgVDAC2 and CgBak in HEK293 cells detected by co-immunoprecipitation. Flag-tagged CgBak and Myc-tagged CgVDAC2 were co-expressed in HEK293T cells. Co-IP was performed with M2 anti-FLAG antibody. Western blot was carried out with anti-Myc antibody. Empty vector was used as negative control. (Top) CgVDAC2 co-immunoprecipitates with CgBak. (Middle and bottom) The expression of CgVDAC2-Myc and CgBak-Flag proteins.

The yeast two-hybrid system was performed to further access the functional relevance of CgVDAC2 and CgBak. A potential interaction between the two proteins was detected, comparing the invisible yeasts (Control) with the blue hybridized yeasts growing on SD/-Ade/-His/-Leu/-Trp plates (Fig 8A). Co-IP was applied to further validate the interaction between CgVDAC2 and CgBak. The results of western blotting showed that the expression of pCMV-N-Myc-CgVDAC in input samples could be detected in the cells of both groups, whereas in IP samples was only detectable in the cells of the control group (Fig 8B), indicating the capability of CgVDAC2 to interact with CgBak in HEK293T cells.

## Discussion

The functional molecular mechanisms of apoptosis system in invertebrates, including oysters, are still largely unexplored. In this study, we identified a gene encoding VDAC2 in *C*. *gigas* (*CgVDAC2*) and analyzed its expression patterns at different developmental stages, in distinct tissues and most importantly under pathogen challenge. The results showed that CgVDAC2 was highly inducible by OsHV-1, co-localized with mitochondria in HeLa cells, suppressed UV irradiation-induced apoptosis and interacted with CgBak. To our knowledge, this is the first report that reveals the immune and apoptotic function of VDAC in mollusks, providing information on the involvement of VDAC in anti-virus immunity and apoptosis modulation.

Three different isoforms of the VDAC family, known as VDAC1, VDAC2 and VDAC3, have been previously reported [36,37]. Many vertebrates have all three types of VDAC isoforms, whereas invertebrates usually have only one type [38]. Although some invertebrates, such as *D*. *melanogaster*, have more than one isoforms, the additional one[s] are derived from the original VDAC through duplication [37]. *CgVDAC2*, the only VDAC found in *Crassostrea gigas*, was predicted to be a VDAC2-like gene, according to annotation information from multiple alignment and phylogenetic analyses. *VDAC2*s are strongly conserved and expressed in an extensive range of organisms from cnidarians to mammals [39]. Phylogenetic analysis showed that *CgVDAC2* was clustered into the group of *VDAC2*, rather than that of *VDAC1*, and was closer to homologs from invertebrates than those from vertebrates. Therefore, *CgVDAC2* belongs to the conserved *VDAC2* family.

VDAC, a fundamental mitochondrial channel protein, has several characteristic motifs that are conserved in all the known eukaryotic homologs [36,40]. Motif scan analysis showed that the coding region of *CgVDAC2* contained a four-element eukaryotic porin signature motif, a GLK motif, and a VKAKV-like sequence (S1 Fig). The GLK motif, a specific amino-acid triplet in VDACs, plays a vital role in ATP nucleotide binding and nucleotide-dependent channel gating [41] and is conserved in VDACs though mutations of the lysine residue, has only limited effect on the functionality of the domain [37]. The VKAKV-like sequence is present in all the available plant and animal *VDAC* sequences [42] and is involved in the insertion of the protein into MOM. BLAST analysis of *CgVDAC2* showed a porin3 domain, which is a typical characteristic of pore-forming proteins [43]. Overall, the results showed that *CgVDAC2* is probably homologous to other *VDAC*s and exhibits similar functions.

Since *VDAC* is constitutively expressed in mitochondria, the decline in the transcription level of *CgVDAC2* between the egg stage and the four-cell stage might indicate the removal of paternal mitochondria from the oocyte during early embryogenesis. In addition, oyster larvae become mobile at the stage of trochophore [44], and the mobile larvae can swim using the developing cilia [45]. The increase in the transcription level of *CgVDAC2* after the trochophore stage (Fig 3A) coincides with the elevated energy demand for movement. The elimination of paternal mitochondria in the oocyte may begin immediately after fertilization and finish approximately at the four-cell stage. Besides, *CgVDAC2* may be associated with the energy supply for movement in the larval stages.

*CgVDAC2* was expressed in almost all the major oyster tissues, indicating its involvement in mitochondrial energy metabolism [46,47]. *CgVDAC2* showed relatively higher expression in the high energy consuming tissues, such as the hemolymph, adductor muscle, digestive gland, and gill (Fig 3B), which are in agreement with previous studies in human, fish, and shrimp [29,48–50]. The higher *CgVDAC2* expression level was observed in the hemolymph and gill, which are the main immunity and respiration tissues in oyster, suggesting that *VDAC*s might also participate in these biological processes. The expression level of *CgVDAC2* was very low in the female gonad, while relatively high in the male gonad (Fig 3B). Similar results that showed differential *VDAC* expression patterns in the male/female gonad have been reported in several other animals. The high expression level of *VDAC* in male reproductive organs is relevant to the important role of this gene in male reproduction processes [51,52], including spermatogenesis, sperm development and maturation, motility, capacitation, and acrosomal reaction [53]. Male mice with *mVDAC3*-deficiency displayed significantly reduced sperm motility and were infertile [54]. As VDAC contains an ATP binding site and mediates ATP transport [55,56], it was suggested that VDAC in the MOM of sperm flagellum is involved in ATP transport and sperm energy metabolism and supplies the required energy for sperm forward motility [53]. Since no *VDAC3* homolog was identified, and *CgVDAC2*, which also contains a putative ATP binding site, was highly expressed in the male gonad, it is possible that *CgVDAC2* participates in the male reproduction of *C*. *gigas*. Previous studies have revealed the involvement of VDACs in viral pathogenesis. *VDAC* is up-regulated upon infection by the *Scophthalmus maximus* rhabdovirus (SMRV) in embryonic cells of olive founder (*Paralichthys olivaceus*) [28]; by grass carp reovirus (GCRV) in grass carp (*Ctenopharyngodon idella*) [49]; and by white spot syndrome virus (WSSV) in kuruma shrimp (*Marsupenaeus japonicus*) [29]. Our studies showed an increased expression of *CgVDAC2* upon infection by OsHV-1, indicating the involvement of *CgVDAC2* in virus-related immunity. However, the mechanisms underlying the VDAC-virus interaction remain unclear. Considering that VDAC participates in energy metabolism, and that the expression level of *VDAC* controls the transport of metabolites between the cytosol and mitochondria [55], while low expression of *VDAC* leads to disrupted energy supplies [26], it is quite possible that once virus impairs the energy metabolism of host cells, the host probably up-regulates the expression of *VDAC* to make up for the loss. In this study, the results of subcellular localization showed the co-localization of CgVDAC2 with mitochondria in HeLa cells, which further suggested the similar function of *CgVDAC2* to its homologs in other animal species and its potential involvement in mitochondrial apoptosis in oyster. So, its involvement in mitochondrial apoptosis seems to be another possible explanation for the induced expression of *CgVDAC2* upon infection by OsHV-1.

Previous studies showed that VDAC takes part in apoptosis, although it remains disputable how this mitochondrial porin functions in apoptotic events [21–23]. VDAC regulates the release of cytochrome c from mitochondria to the cytosol in mitochondria-mediated apoptosis, which leads to caspase3 activation and apoptosis; however, these results are still controversial, since they have been challenged by some studies [57–59]. VDAC regulates apoptosis mainly by its interaction with Bax and Bak, two pro-apoptosis proteins of the Bcl-2 family [25,60]. Despite the disagreement [60–62], most studies have suggested that this interaction suppresses the functions of Bax and Bak, and thus inhibits the cytochrome c release and apoptosis effects. However, previous studies on the apoptosis regulation function of VDAC have mainly focused on mammals. In this study, we found that the hemocytes of the Pacific oyster with RNAi-silenced *CgVDAC2* had higher apoptosis rates. These results suggested the participation of *CgVDAC2* in the apoptosis process of *C*. *gigas* and were consistent with those reported in mammals, in which the deletion of *VDAC2* led to a higher apoptosis rate in the mouse embryo fibroblast (MEF) [25] and a rapid apoptosis in the mouse lymphocyte [63]. Besides, previous studies on mammals have demonstrated that VDAC2 forms a specific protein complex with Bak and inhibits the pro-apoptotic function of the latter [25,64]. Additionally, it has been shown that the rapid apoptosis caused by the deletion of *VDAC2* in mouse lymphocytes can be reversed with the deletion of *Bak* [63]. Our results showed that while CgBak, the homolog of Bak in *C*. *gigas*, was able to enhance the activity level of caspase3 in UV irradiated HEK293T cells, co-overexpression of CgVDAC2 with CgBak could reduce the activity of caspase3 to a relatively lower level than that in the former case. We also identified the interaction between CgVDAC2 and CgBak *in vitro* using the yeast two-hybrid system and co-IP assay. Taken together, all the results suggest that CgVDAC2 might involve in oyster apoptosis by interacting with and suppressing the activation of CgBak. Although limited information is available on the mechanism underlying the mitochondrial-mediated apoptotic pathway in bivalve mollusks, studies in *Mytilus galloprovincialis* [65], *Crassostrea hongkongensis* [6] and *Hydra magnipapillata* [66] on the apoptosis-related function of Bak, in varying extent, suggested the pro-apoptotic functions of Bak in the respective species, which are consistent with the functions of their homologous proteins in vertebrates and further implied the possible pro-apoptotic function of *CgBak*. In summary, *CgVDAC2* might interact with and suppress the activation of *CgBak*, which probably resulted in the inhibition of apoptosis in oyster.

Since oyster cell lines and reverse genetic methods are not widely available, we used mammal cells in several assays as molecular tools, which is a typical way especially for the study of species without established cell lines [66–68]. The results of this study may help to better understand the role of CgVDAC2 in immunity and apoptosis, despite the use of HEK293T cells and HeLa cells instead of oyster cells.

Apoptosis is an irreplaceable part of the defense system in oyster [4,7,8], which helps it to scavenge the infected cells and restrain the spread of pathogenic organisms. Hence, the inhibition of host cell apoptosis is a critical step for a number of pathogenic organisms. Throughout the process of pathogen-host co-evolution, viruses have acquired distinct strategies to inhibit host cell apoptosis [69]. It is known that Epstein-Barr virus (EBV) leads to a down-regulation of *Bim* (a pro-apoptotic Bcl-2 family member) in infected human B-lymphocytes, reducing their propensity to apoptosis [70,71]. *Perkinsus marinus*, an intracellular protozoan that causes Dermo disease and extensive mortalities in oysters, is capable to suppress apoptosis and freely proliferate and spread in the host cell [10]. Upon infection by WSSV, the expression levels of *MjVDAC* increase in kuruma shrimp, while *MjVDAC* knockdown delays the infection [29]. In *C*. *gigas*, *CgVDAC2* was up-regulated upon infection by OsHV-1, leading to the inhibition of apoptosis that is responsible to eliminate the infected cells. This study provided insight into the immune and apoptotic functions of CgVDAC2 and the defense system of *C*. *gigas* that will aid in the development of virus control and disease prevention measures in aquaculture.

## Supporting Information

S1 Fig cDNA and deduced amino acid sequences of *CgVDAC2*. Nucleotides and amino acids are numbered on the left. The four-element eukaryotic porin signature motif is shaded; the GLK motif is underlined; and the VKAKV-like sequence is boxed.
(TIF)

S2 Fig Multiple alignment of *VDAC2* in *Crassostrea gigas* and homologs in other species obtained from GenBank. '*', ':', and '.' indicate positions with single, strongly, and weakly conserved residues, respectively.
(TIF)
