# Supplementary figures and images for: Characterization of Oyster Voltage-Dependent Anion Channel 2 (VDAC2) Suggests Its Involvement in Apoptosis and Host Defense
Source: PLoS One. 2016 Jan 4;11(1):e0146049. doi: 10.1371/journal.pone.0146049 (PMC4700975; doi:10.1371/journal.pone.0146049)

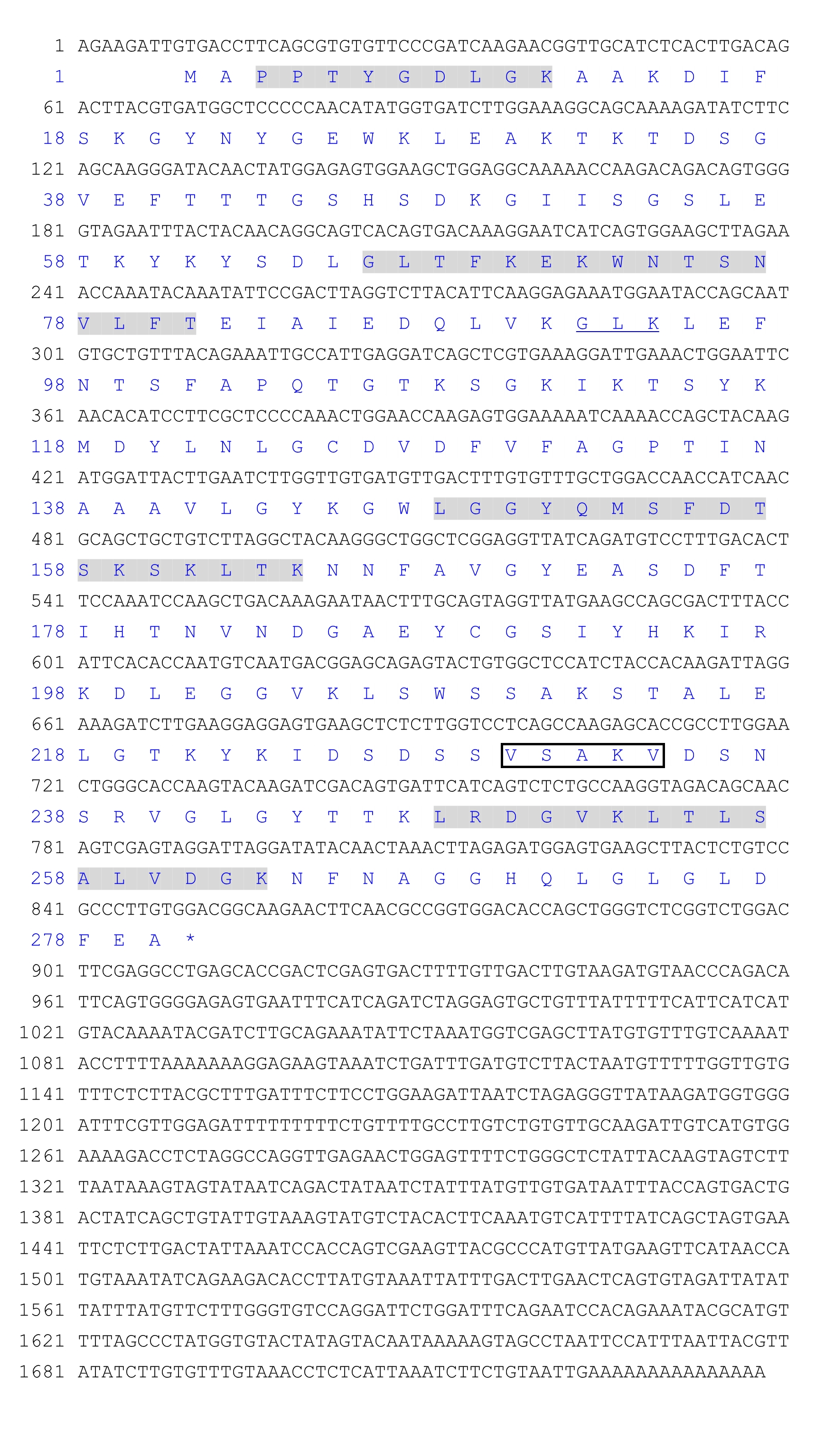

Supplement: S1 Fig — Nucleotides and amino acids are numbered on the left. The four-element eukaryotic porin signature motif is shaded; the GLK motif is underlined; and the VKAKV-like sequence is boxed. (TIF) [file pone.0146049.s001.tif]

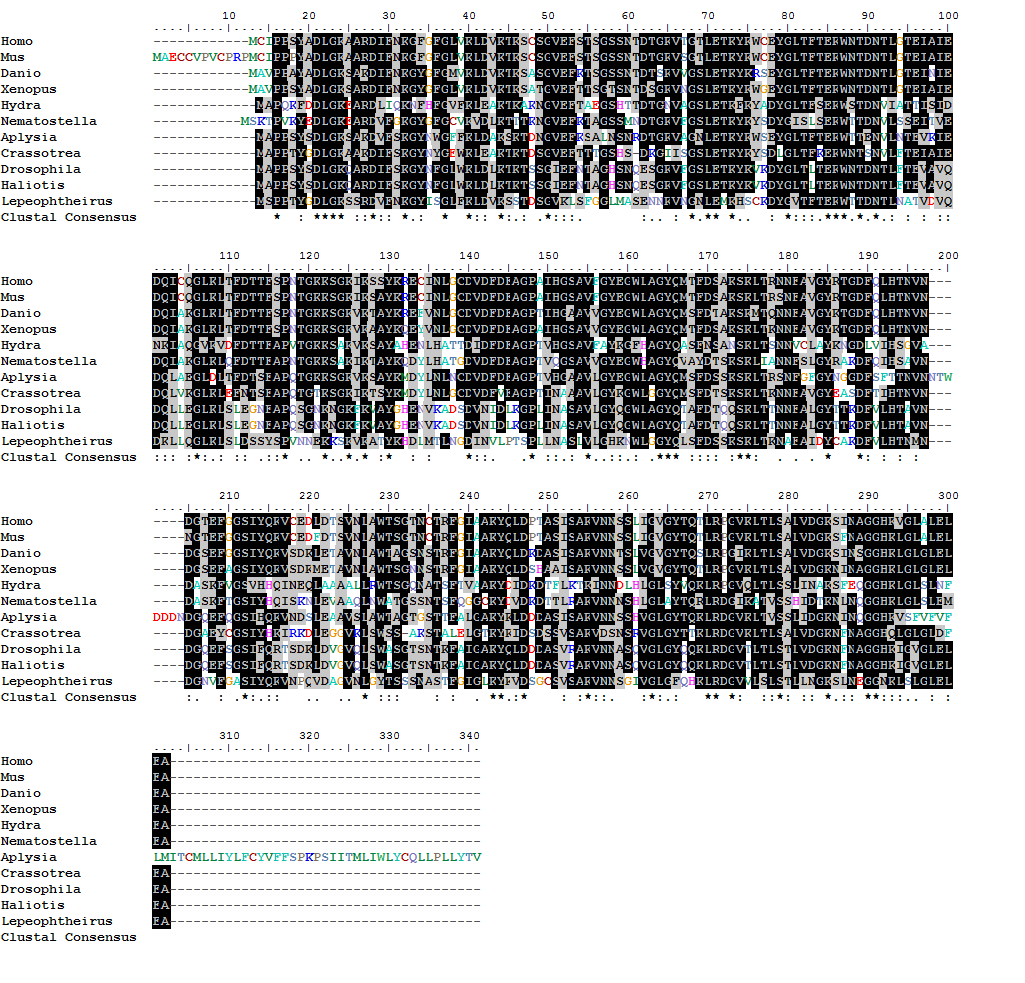

Supplement: S2 Fig — '*', ':', and '.' indicate positions with single, strongly, and weakly conserved residues, respectively. (TIF) [file pone.0146049.s002.tif]
